# Comparison of Shear Bond Strength and Estimation of Adhesive Remnant Index Between 3D Printed Customised Lingual Buttons and Prefabricated Lingual Buttons: An In Vitro Study

**DOI:** 10.7759/cureus.106753

**Published:** 2026-04-09

**Authors:** Jacob John, Veena Nagappan Kamalabhai, Salman Amir, Jibin J Daniel, Navin Oommen Thomas, Nevin Abraham

**Affiliations:** 1 Department of Orthodontics and Dentofacial Orthopaedics, Pushpagiri College of Dental Sciences, Thiruvalla, IND; 2 Department of Orthodontics and Dentofacial Orthopaedics, Sri Sankara Dental College, Thiruvananthapuram, IND

**Keywords:** 3d printing, adhesive remnant index, customization, direct bonding, lingual buttons, orthodontics, shear bond strength

## Abstract

Aim

The main aim of this study is to compare the shear bond strength (SBS) of 3D-printed customised lingual buttons and prefabricated lingual buttons.

Methodology

Forty-eight extracted maxillary premolar teeth were selected. Teeth were allocated to two groups of 24 each; in one group, customised lingual buttons were fabricated using 3D printing. Prefabricated lingual buttons were bonded to one group, while the other group was bonded with the customised lingual buttons. The samples were preserved in distilled water at 37⁰C for 24 hours. The samples were evaluated for SBS using a universal testing machine, and the adhesive remnant index (ARI) was determined under a stereomicroscope at 10× magnification. Statistical analysis included the Mann-Whitney U test for SBS and the chi-square test for ARI scores.

Results

The mean SBS of prefabricated and customised 3D-printed lingual buttons was estimated to be 2.331 ± 2.549 MPa and 3.694 ± 2.564 MPa, respectively. The Mann-Whitney U test conducted to analyse the SBS showed a significant difference between the two groups at p < 0.05. ARI scores indicated that prefabricated lingual buttons had an ARI score of 3 predominantly, while customised 3D-printed lingual buttons had a predominant score of 1. This was statistically significant.

Conclusion

Customised 3D-printed lingual buttons showed superior SBS compared to the prefabricated lingual buttons. Bond failure was noted more at the bracket-adhesive interface in the prefabricated lingual button group, while it was a cohesive failure close to the enamel surface in the case of customised lingual buttons.

## Introduction

Three-dimensional printing is a process that creates an object by adding material in a specific manner, layer by layer [1]. It is also known as additive manufacturing, since it develops physical products from digital design files by joining or forming input substrate material using a layer-upon-layer printing technique [2,3]. Initially, the technique of 3D printing used was stereolithography, with standard tessellation language (STL) format [4]. The STL file is the most commonly used file format for editing and preparing objects for 3D printing. Since then, various cutting-edge technologies, like fused deposition modelling (FDM), PolyJet photopolymer (PPP) printing, selective laser melting (SLM), and direct metal laser sintering (DMLS), have been successfully introduced in 3D printing [5].

With new-generation printers rendering accurate and affordable prototypes, 3D printing has proven economical and easy to use in orthodontic practice. Orthodontic brackets and auxiliaries, such as retraction hooks, molar turbos, lingual buttons, and customized appliances like the Herbst appliance, have been fabricated with a 3D printer. The advantage of 3D printing is that everything is customizable [6]. In contemporary orthodontics, bonding with composite adhesive is the most commonly used method to fix attachments over tooth surfaces using adhesive resins. Direct bonding of orthodontic brackets, molar tubes, and lingual buttons was considered one of the most crucial developments in this domain. Earlier, banding was used, but it was tedious, unaesthetic, and most uncomfortable for the patient. Proper hygiene maintenance by patients was difficult, and orthodontists faced many disadvantages [7]. Now, most orthodontists bond attachments, like brackets, buttons, and tubes, directly or indirectly over the tooth surface [8].

Bond strength depends on various factors, like bracket base, mesh design, and characteristics of adhesive resins [9]. Among the different factors, the close adaptation of the bracket base to the tooth surface could be one of the significant factors determining bond strength. Since the brackets, lingual buttons, and molar tubes available in the market are prefabricated, and the tooth morphology in each individual differs, adaptability also differs. With the advent of various 3D technologies in orthodontics, customization for each patient is possible. Three-dimensional printing also increases precision and accuracy [10]. Since brackets, buttons, and multiple attachments can be custom-made based on each patient's tooth morphology, a close adaptation between the tooth and attachments may be achieved. The cost factor is also decreasing, as many orthodontists now use in-house printers. We hypothesize that bond strength could be improved with the customization achieved through 3D printing.

In order to verify our hypothesis, we conducted a study using 3D-printed customized lingual buttons, which were fabricated using SLM technology. Lingual buttons are accessory attachments usually placed on the lingual aspect of teeth. They are commonly used for correction of rotations [11], closure of relapsed spaces [12], and interception of lip-sucking habits [13]. SLM technology/direct metal laser melting (DMLM), used in our study, is one of the many powder-bed fusion technologies. In this process, a high-energy laser beam selectively melts metallic powder particles layer by layer, according to a computer-aided design (CAD) model, resulting in the fabrication of highly precise 3D structures [5]. Unlike selective laser sintering, where particles are partially fused, SLM achieves complete melting of the powder, producing dense components with superior mechanical properties and structural integrity. The SLM process involves spreading a thin layer of metal powder over a build platform, followed by selective melting using a laser beam guided by digital data. After each layer is fused, the platform is lowered, and a new layer of powder is applied. This layer-by-layer fabrication continues until the final structure is completed [14].

In dentistry, SLM has gained significant attention due to its high precision, customization capability, and ability to fabricate durable metal components. It has been widely used in implantology for producing patient-specific titanium meshes and implant frameworks, as well as in prosthodontics for crowns, bridges, and removable partial denture frameworks. Within orthodontics, 3D printing technologies, including SLM, have enabled the fabrication of customized appliances, such as brackets, lingual systems, retainers, indirect bonding trays, and skeletal anchorage devices. These technologies facilitate improved fit, enhanced accuracy, and better treatment planning through digital workflows [11,12].

In our study, the bond strength of the customized lingual button was compared with prefabricated lingual buttons, which were available in the market, to determine whether the close adaptability of the button to the tooth surface would increase bond strength.

## Materials and methods

The study was approved by the Institutional Ethics Committee (PCDS/IEC/J12/11/16). Extracted premolar teeth were obtained and debrided thoroughly. They were then examined for caries, pre-existing fractures, restorations, fluorosis, and demineralisation. The teeth were categorised into two groups of 24 each and were cleaned and preserved in 0.1% aqueous thymol solution. Extracted maxillary first premolar teeth were collected from the Department of Oral and Maxillofacial Surgery, Pushpagiri College of Dental Sciences, Thiruvalla, Kerala, India. Teeth were preserved for two months before use.

Fabrication of 3D printed lingual buttons

**Figure 1:**
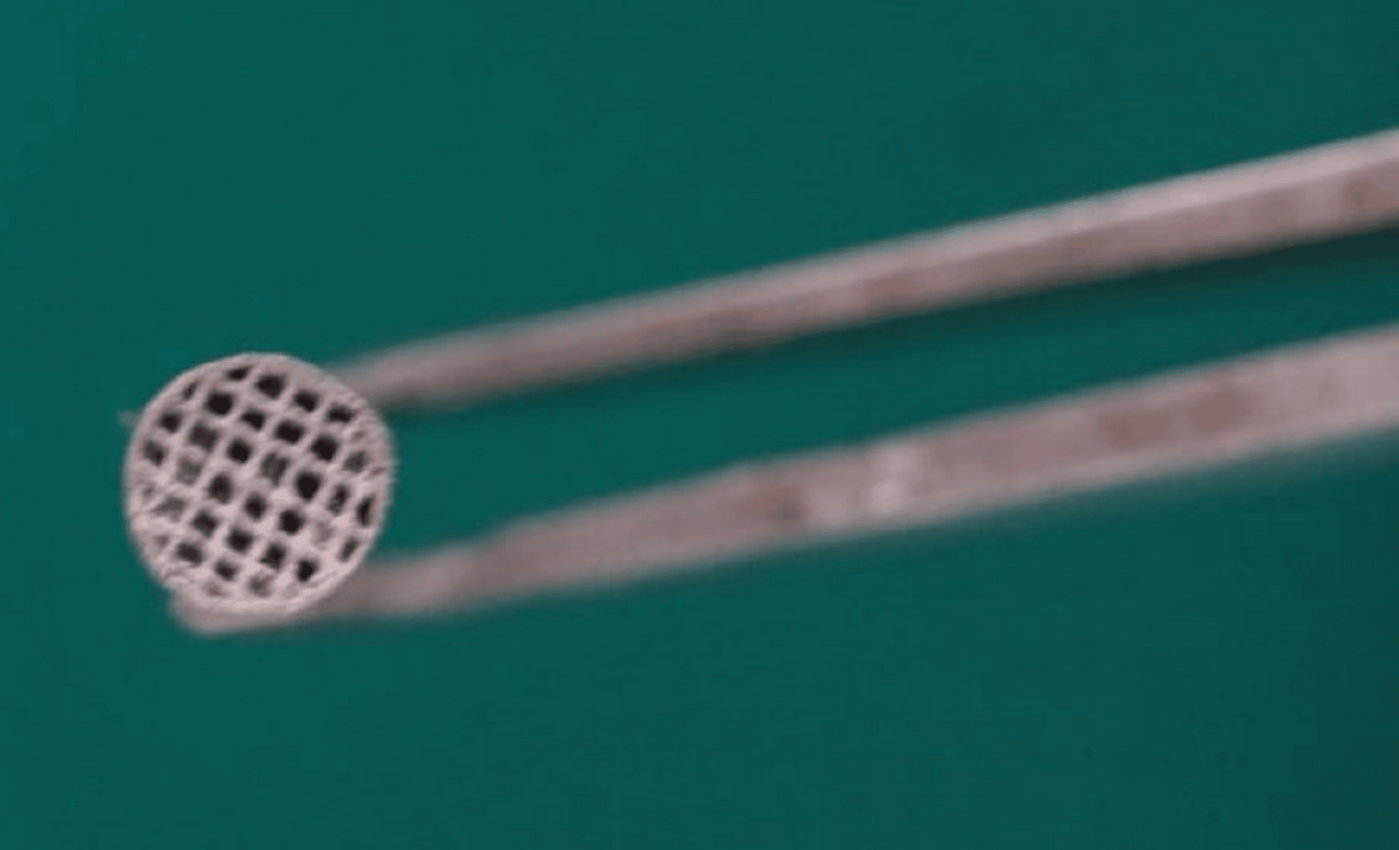
Customised 3D Printed Lingual Buttons

The 3D scanning of the palatal surface of each selected tooth was done using a desktop dental scanner (MEDIT T 500, Seongbuk-gu, Seoul, Korea). The STL file of the lingual button, with the base adapted for each tooth, was designed using R&D 3D-modelling software developed by Dentcare Dental Lab, Muvattupuzha, Kerala. The lingual button mesh was designed similarly to that of the prefabricated ones. The customised lingual buttons were fabricated using SLM technology, involving the following processes (Figure 1) [14].

A program for 3D printing using the STL file was created in the computer software Magic Software, version 24.01, developed in Belgium. The program was then loaded into the 3D printer (SLM Solutions SLM 280 HL, Lübeck, Germany). After loading the program, the baseplate (metal plate on which metal powder was deposited and then fused) was placed in the 3D printing chamber. The next process was flooding. Flooding is the process of removing oxygen from the printing chamber. The oxygen level should be below 0.5% for 3D printing. The temperature of the baseplate was adjusted to 75°C, which was optimal for the 3D printing of stainless steel parts. Stainless steel 316L (0.03% carbon) was used for the study. Then, a layer of powder was coated onto the baseplate. This process is called layer adjustment for proper adhesion. Initial exposure to laser light was then done. The powder was laser-melted and layered until the entire structure of the lingual button was completed. Post-fabrication finishing was done by sandblasting.

Bonding procedure

The palatal enamel surface of each tooth was etched with 37% orthophosphoric acid gel (3M™ Scotchbond™ Universal Etchant Etching Gel, Monrovia, CA, USA) for 30 seconds, and thoroughly rinsed with a water spray for 30 seconds. The tooth was gently dried to obtain the chalky white appearance.

A thin coat of light-cure adhesive, Transbond XT primer (3M Unitek light-cure adhesive primer, Monrovia, CA, USA), was applied to the acid-etched enamel surface. Each 3D-printed customised lingual button was placed on the corresponding tooth. This meant that each customised lingual button was specific to each tooth. Transbond™ XT (3M Unitek light-cure adhesive composite, Monrovia, CA, USA) was then applied to the lingual button base, which was placed and pressed against the palatal surface of the tooth with a force of ≈2.94 N. Three-dimensional printed lingual buttons were placed on the specific tooth. Excess composite (flash) was removed using a probe. Bonded buttons were cured for 40 seconds, utilising a light-curing unit (LED.D 800, 2 mW/cm², Ivoclar Vivadent, Schaan, Liechtenstein).

Shear bond strength (SBS) estimation

All specimens were preserved in distilled water for 24 hours before the SBS test. The specimens were placed in a custom-made mounting jig in the universal testing machine so that the base of the lingual button was parallel to the shear-peel load. Each tooth was assigned a number to anonymise the data, ensuring that the outcome assessor was blinded. A shear debonding force was applied to the lingual button in an occlusogingival direction, at a crosshead speed of 0.5 mm/min. The maximum force necessary to debond, or the initial button fracture, was recorded in Newtons (N) and then converted into megapascals (MPa) using the formula: $\text{SBS (MPa)} = \frac{\text{Peak load at bond failure (N)}}{\text{Button surface area (mm}^2\mathrm{)}}$.

The average radius and anvil height of the lingual buttons were entered into the universal testing machine, which estimated the average surface area. The surface area of the prefabricated lingual button was 9.19 mm². The average surface area of the 3D-printed lingual button was estimated to be 9.62 mm².​​​​​​

Test for debonding characteristics

The tooth surface of debonded samples was studied under 10× magnification in a stereo microscope (Mantis, Vision Engineering, Watertown, CT, USA). The debonding characteristics were assessed using the adhesive remnant index (ARI), which represents the amount of residual adhesive on the tooth surface [15]. Score 0 denotes no adhesive on the tooth; Score 1 indicates less than half of the adhesive remaining on the tooth; Score 2 indicates more than half of the adhesive remaining on the tooth; and Score 3 implies the complete presence of adhesive on the tooth, with a clear impression of the bracket mesh.

Statistical analysis

The data were analysed using IBM SPSS Statistics for Windows, Version 21 (Released 2012; IBM Corp., Armonk, NY, USA). Descriptive statistics of SBS were calculated. The normality of the data for SBS was determined using the Shapiro-Wilk test. Since the data were not normally distributed, the Mann-Whitney U test (95% confidence interval) was used to determine the difference in SBS between the prefabricated and 3D-printed lingual button groups. The chi-square test was performed to determine the differences in ARI between the two groups.

## Results

Table 1 illustrates the descriptive statistics for the SBS of the two groups. The Mann-Whitney U test revealed a significant difference between the two groups (Table 2).

**Table 1:** Descriptive Statistics for SBS SBS: shear bond strength

Group	N	Mean	SD	Std. error mean	95% confidence interval for mean lower bound	95% confidence interval for the mean upper bound	Minimum	Maximum
Group 1 prefabricated buttons	24	2.331232	2.5495796	0.5204308	1.25	3.40	0.065	11.80
Group 2 3D printed	24	3.694467	2.5643566	0.5234471	2.61	4.77	0.218	9.87

**Table 2:** Mann-Whitney U Test for SBS Between Groups SBS: shear bond strength

	Mann-Whitney U	Wilcoxon W	Z	p
Between group 1 and group 2	179.000	479.000	-2.248	0.025

Adhesive remnant index (ARI)

Cross-tabulation shows ARI Score 3 being predominant in Group 1 (prefabricated lingual buttons), while ARI Score 1 is predominant in Group 2 (3D-printed lingual buttons) (Table 3). Group 1 has 0% of ARI Score 0, 29.2% of ARI Score 1, 25.0% of ARI Score 2, and 45.8% of ARI Score 3; Group 2 has 20.8% of ARI Score 0, 54.2% of ARI Score 1, 16.7% of ARI Score 2, and 8.3% of ARI Score 3. The chi-square test was performed to determine the differences in ARI scores between the two groups. It was found that there was a statistically significant difference (p < 0.05) between the two groups in ARI scores, with a chi-square value of 0.004 (Table 4).

**Table 3:** Cross Tabulation for ARS Score ARS: adhesive remnant index

ARS score group cross-tabulation					
			Groups		Total
			Group 1	Group 2	
ARS score	0.00	Count	0	5	5
		% within ARS score	0.0%	100.0%	100.0%
		% within group	0.0%	20.8%	10.4%
	1.00	Count	7	13	20
		% within ARS score	35.0%	65.0%	100.0%
		% within group	29.2%	54.2%	41.7%
	2.00	Count	6	4	10
		% within ARS score	60.0%	40.0%	100.0%
		% within group	25.0%	16.7%	20.8%
	3.00	Count	11	2	13
		% within ARS score	84.6%	15.4%	100.0%
		% within group	45.8%	8.3%	27.1%

**Table 4:** Chi-Square Tests for the ARI Score of Two Groups * denotes statistically significant ARS: adhesive remnant index

	Value	df	Asymp Sig. (two-sided)
Pearson chi-square	13.431	3	0.004*
Likelihood ratio	16.022	3	0.001
Linear-by-linear association	13.027	1	0.000
No. of valid cases	48		

## Discussion

In fixed orthodontic treatment, the bond failure rate has been shown to be about 4%-10% [16,17]. Studies have shown that factors such as enamel conditioning, bracket base design, adhesive system type, bonding technique, and mode of cure can influence the SBS [9]. Another determining factor could be how closely the bonding mesh base of the prefabricated attachment is adapted to the teeth. Tooth morphology varies significantly between individuals, and attaining a uniform attachment base adaptation to the tooth is virtually impossible with prefabricated attachments. However, with the advent of 3D printing technology in orthodontics, customisation depending on the tooth form and shape is now possible.

In the literature, there are case reports regarding various customised orthodontic appliances, such as the eruption guidance appliance, molar distaliser, C-palatal plate, and band-and-loop space maintainer. These case reports have found that treatment results using the customised appliances were excellent [18-21]. Comparative studies have shown that 3D-printed retainers had a proper fit compared to conventional retainers, which increased accuracy and reliability [22]. Similarly, the 3D-printed band-and-loop retainer had a superior fit and more accurate details compared to conventional ones [21]. Sha et al. [22] conducted a study on lingual indirect bonding systems and demonstrated that CAD/CAM (computer-aided manufacturing)-based customised bracket systems had a debonding force that was higher than or equal to that of the conventional bracket system.

To our knowledge, there is no relevant study in peer-reviewed journals assessing whether customisation helps improve bond strength in direct bonding techniques. Hence, the current study was designed to compare the SBS between the prefabricated and 3D-printed lingual buttons. The study also assessed the interface at which bond failure had occurred. In our study, 3D-printed lingual buttons had a mean SBS of 3.694 ± 2.564, while the prefabricated lingual buttons showed a mean bond strength of 2.331 ± 2.549. Alkhateeb and Al-Sheakli [23] contrasted the SBS of lingual buttons with round and rectangular bases. They found that lingual buttons with rectangular bases had lower SBS, as the mesiodistal dimension of the button did not correctly fit the palatal contour of the tooth. Similarly, the superior SBS of the 3D-printed lingual buttons compared to the prefabricated ones could probably be due to the custom fit of the lingual button base to the tooth surface in this study.

The ARI was measured to assess the interface at which bond failure occurs. In the present study, the prefabricated lingual buttons had a predominant score of 3, which showed that bond failure occurred at the bracket/adhesive interface, thus reducing the possibility of enamel trauma. Three-dimensional printed lingual buttons had a predominant score of 1, showing bond failure within the adhesive. According to Sharma-Sayal et al. [24], the bracket base/adhesive interface has been the weak link in orthodontic bonding. Our study results demonstrate that customisation with 3D printing can overcome this issue. While it has been proposed that increased residual adhesive mitigates enamel fracture, it is evident that lesser residual adhesive on the tooth is beneficial for professionals, as it conserves time and diminishes the risk of enamel damage throughout the cleaning procedure [16].

O'Brien et al. [25] determined that the ARI score was influenced by various aspects, notably the bracket base configuration, the adhesive type, and not solely the strength of bonding at interfaces. Further investigations in this regard should be attempted. In our study, customised lingual buttons were fabricated using the technique of SLM. Different techniques of 3D printing, such as selective laser sintering, SLM, selective heat sintering, and DMLS, can be used to fabricate metal parts. This approach was chosen due to its benefits, including the capacity to adjust attributes during component processing, enhanced functionality, a relatively affordable price, and the manufacture of near-net-shaped components suitable for application [26]. The metallic components produced using SLM technology demonstrate superior quality and display commendable performance [27].

The lingual buttons were affixed with a fourth-generation composite adhesive because of its superior long-term clinical performance and its status as the most widely utilised bonding technology globally. The fourth-generation glue system is economically efficient while maintaining equivalent binding strength. It is applicable for nearly all bonding protocols: direct, indirect, self-cure, dual-cure, or light-cure. These systems remain the benchmarks against which newer systems are evaluated [28]. The samples underwent testing with a universal testing machine to ascertain the SBS. The primary benefit of a universal testing machine is its ability to accommodate various sample dimensions and designs, thereby facilitating diverse testing procedures. The universal testing machine can detect force variations as minimal as 0.001 g. The equipment has undergone comprehensive testing and is widely recognised as a standard device to determine parameters such as tensile strength, friction between brackets and archwire, and bond strength. The mean SBS obtained for both groups was less than that obtained in previous studies [23,29]. Many factors, such as the acid etching technique, curing light intensity, curing time, adhesive system, and bracket base design, affect the SBS [16-18]. Further extensive studies must be conducted to evaluate the influence of these factors.

Limitations, generalisability, and further studies

The limitation of the present study is that, as an in-vitro investigation, it cannot fully replicate the intricate oral processes. In vivo, complicated interactions encompass intra-oral ageing of resin composites, pH variations, temperature shifts, intricate cyclic loading, microbial invasion, and enzymatic destruction. The research was conducted on extracted teeth from the Keralite population. Given the promising outcomes of the current study, additional in vivo research with diverse populations and larger sample sizes should be undertaken.

## Conclusions

Within the constraints of this investigation, the subsequent conclusions can be inferred: customised 3D-printed buttons had a greater SBS compared to prefabricated lingual buttons. Prefabricated lingual buttons exhibited bond failure mostly at the bracket/adhesive interface, whereas customised 3D-printed lingual buttons showed major cohesive bond failure near the tooth surface.

## References

[1] Dawood A, Marti Marti B, Sauret-Jackson V, Darwood A (2015). 3D printing in dentistry. Br Dent J.

[2] Horn TJ, Harrysson OL (2012). Overview of current additive manufacturing technologies and selected applications. Sci Prog.

[3] Choonara YE, du Toit LC, Kumar P, Kondiah PP, Pillay V (2016). 3D-printing and the effect on medical costs: a new era?. Expert Rev Pharmacoecon Outcomes Res.

[4] Whitaker M (2014). The history of 3D printing in healthcare. Bull R Coll Surg Engl.

[5] Shahrubudin N, Lee TC, Ramlan R (2019). An overview on 3D printing technology: technological, materials, and applications. Procedia Manufacturing.

[6] Kravitz ND, Groth C, Shannon T (2018). CAD/CAM software for three-dimensional printing. J Clin Orthod.

[7] Yadav J, Mehrotra P, Kapoor S, Mehrotra R (2013). Basis of orthodontics-bonding - a review. Int J dent Sci Res.

[8] Nawrocka A, Lukomska-Szymanska M (2020). The indirect bonding technique in orthodontics - a narrative literature review. Materials (Basel).

[9] Bakhadher W, Halawany H, Talic N, Abraham N, Jacob V (2015). Factors affecting the shear bond strength of orthodontic brackets - a review of in vitro studies. Acta Medica (Hradec Kralove).

[10] Nguyen T, Jackson T (2018). 3D technologies for precision in orthodontics. Semin Orthod.

[11] Yezdani AA (2013). Composite lingual buttons for correction of rotated premolar teeth. Int J Orthod Milwaukee.

[12] Fayyaz Ahamed S (2013). Closure of relapsed spaces with lingual buttons. J Clin Orthod.

[13] Durgekar SG, Naik V (2009). Lingual buttons to intercept lip-sucking habits. J Clin Orthod.

[14] Song X, Zhai W, Huang R, Fu J, Fu MW, Li F (2020). Metal-based 3D-printed micro parts & structures. Encycl Mater: Met Alloys.

[15] Artun J, Bergland S (1984). Clinical trials with crystal growth conditioning as an alternative to acid-etch enamel pretreatment. Am J Orthod.

[16] Murfitt PG, Quick AN, Swain MV, Herbison GP (2006). A randomised clinical trial to investigate bond failure rates using a self-etching primer. Eur J Orthod.

[17] Naqvi ZA, Shaikh S, Pasha Z (2019). Evaluation of bond failure rate of orthodontic brackets bonded with green gloo-two way color changes adhesive: a clinical study. Ethiop J Health Sci.

[18] Kook YA, Lim HJ, Park JH, Lee NK, Kim Y (2021). 3D digital applications of the modified C-palatal plate for molar distalization. J Clin Orthod.

[19] Graf S, Vasudavan S, Wilmes B (2020). CAD/CAM metallic printing of a skeletally anchored upper molar distalizer. J Clin Orthod.

[20] Khanna S, Rao D, Panwar S, Pawar BA, Ameen S (2021). 3D printed band and loop space maintainer: a digital game changer in preventive orthodontics. J Clin Pediatr Dent.

[21] Cole D, Bencharit S, Carrico CK, Arias A, Tüfekçi E (2019). Evaluation of fit for 3D-printed retainers compared with thermoform retainers. Am J Orthod Dentofacial Orthop.

[22] Sha HN, Choi SH, Yu HS, Hwang CJ, Cha JY, Kim KM (2018). Debonding force and shear bond strength of an array of CAD/CAM-based customized orthodontic brackets, placed by indirect bonding - an in vitro study. PLoS One.

[23] Alkhateeb HM, Al-Sheakli EI (2013). Shear bond strength of different lingual buttons bonded to wet and dry enamel surfaces with resin-modified glass ionomer cement: in vitro comparative study. J Baghdad Coll Dent.

[24] Sharma-Sayal SK, Rossouw PE, Kulkarni GV, Titley KC (2003). The influence of orthodontic bracket base design on shear bond strength. Am J Orthod Dentofacial Orthop.

[25] O'Brien KD, Watts DC, Read MJ (1988). Residual debris and bond strength - is there a relationship?. Am J Orthod Dentofacial Orthop.

[26] Gokuldoss PK, Kolla S, Eckert J (2017). Additive manufacturing processes: selective laser melting, electron beam melting and binder jetting-selection guidelines. Materials (Basel).

[27] Gunasekaran J, Sevvel P, Solomon IJ (2021). Metallic materials fabrication by selective laser melting: a review. Mater Today Proc.

[28] Sofan E, Sofan A, Palaia G, Tenore G, Romeo U, Migliau G (2017). Classification review of dental adhesive systems: from the IV generation to the universal type. Ann Stomatol (Roma).

[29] Oz AA, Yazicioglu S, Arici N, Akdeniz BS, Murat N, Arıcı S (2013). Assessment of the confidence of the adhesive remnant index score with different methods. Turkish J Orthod.

